# Coronary artery size and origin imaging in children: a comparative study of MRI and trans-thoracic echocardiography

**DOI:** 10.1186/1532-429X-16-S1-P110

**Published:** 2014-01-16

**Authors:** Tarique Hussain, Sujeev Mathur, Reza Razavi, Rene Botnar, John M Simpson, Gerald F Greil

**Affiliations:** 1 Imaging Sciences, 4th Floor Lambeth WIng, King's College London, London, UK; 2 Evelina London Children's Hospital, London, UK

## Background

Echocardiographic imaging becomes progressively more difficult beyond infancy. The purpose of this study was to see how coronary magnetic resonance angiography (CMRA) compared to echocardiography for the detection of coronary artery origins and to compare CMRA measurements for coronary dimensions in children with published echocardiographic reference values

## Methods

Any child undergoing a clinical cardiovascular MRI under general anesthesia was included in this study. Enrolled patients underwent dual phase CMRA and echocardiography under the same anesthetic. The dual phase (end-systole and mid-diastole) approach has been recently shown to improve coronary imaging by providing the ability to retrospectively select the optimum phase. Echocardiographic measurements of the right coronary artery (RCA), left anterior descending (LAD) and left main (LM) were made from inner edge to inner edge, excluding points of branching. LM was measured at its mid-point. The LAD & RCA were measured 0.2 to 0.5 mm from its origin. CMRA dimensions were assessed manually at the same points as the echocardiographic measurements. The number of proximal LAD branches imaged was also recorded in order to give an estimate of distal coronary tree visualization.

## Results

50 patients (24 boys, mean age 4.0 years (range 18 days-18 years)) underwent dual-phase CMRA. Coronary origins were identified in 47/50 cases for CMRA (remaining 3 were infants aged 3 months, 9 months and 11 months). In comparison, origins were identified in 41/50 cases for echo (remaining were all older children). Six children had abnormal coronary origins. Five were identified by CMRA and the remaining one was classified as unsure (3/12 age). Only four were identified on echocardiogram. The remaining two, incorrectly classified as normal, had abnormal coronary origins shown at surgery. CMRA performed better than echocardiography in terms of distal visualization of the coronary tree (median 1 LAD branch (IQR 0-2) vs. 0 (IQR 0-1); p = 0.001 by Wilcoxon-Signed-Ranks test). Bland-Altman plots show a systematic bias between echocardiography and CMRA for coronary measurements. CMRA measurements did vary according to cardiac phase. A repeated measures model demonstrates that the systolic coronary dimension (estimated marginal mean 1.90 ± 0.05 mm) is greater than the diastolic measurement (estimated marginal mean 1.84 ± 0.05 mm (p = 0.002).

## Conclusions

Dual-phase CMRA has an excellent (94%) success rate for the detection of coronary origins in children. CMRA measurements have a systematic bias when compared to echocardiographic measurements. Future coronary reference values should quote the systolic diameter. Newborn infants remain challenging and echocardiography remains the modality of choice for this age group.

## Funding

This research was supported by the National Institute for Health Research (NIHR) Biomedical Research Centre at Guy's and St Thomas' NHS Foundation Trust and King's College London. The views expressed are those of the author(s) and not necessarily those of the NHS, the NIHR or the Department of Health.

**Figure 1:**
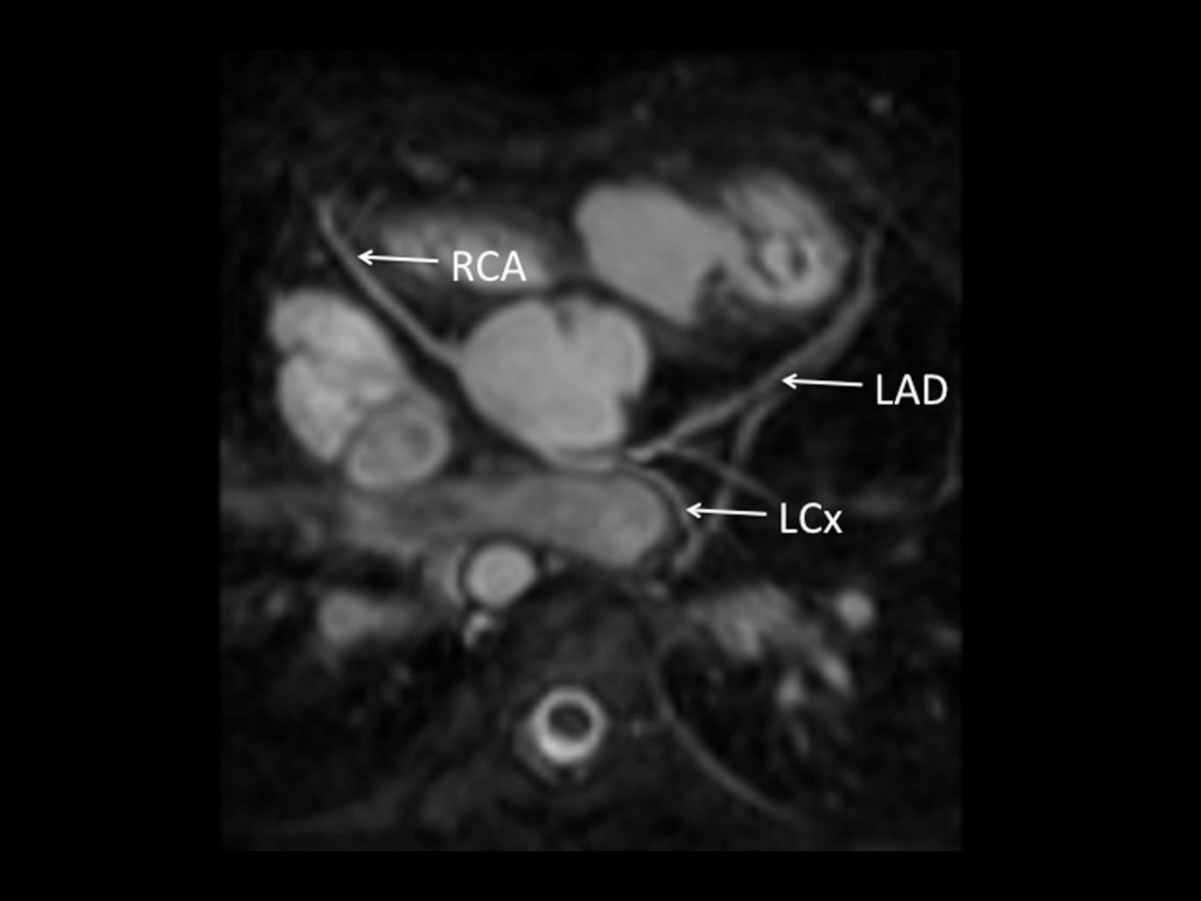
**7 year old girl with repaired pulmonary atresia and ventricular septal defect**. RCA arises from posterior non-coronary cusp. Also seen on echocardiogram

